# Development of a novel hypoxia-immune–related LncRNA risk signature for predicting the prognosis and immunotherapy response of colorectal cancer

**DOI:** 10.3389/fimmu.2022.951455

**Published:** 2022-09-14

**Authors:** Likun Luan, Youguo Dai, Tao Shen, Changlong Yang, Zhenpu Chen, Shan Liu, Junyi Jia, Zhenhui Li, Shaojun Fang, Hengqiong Qiu, Xianshuo Cheng, Zhibin Yang

**Affiliations:** ^1^ Department of Gastric and Intestinal Surgery, The Third Affiliated Hospital of Kunming Medical University/Yunnan Tumor Hospital, Kunming, China; ^2^ Department of Colorectal Surgery, The Third Affiliated Hospital of Kunming Medical University/Yunnan Tumor Hospital, Kunming, China; ^3^ Tumor Institute, The Third Affiliated Hospital of Kunming Medical University/Yunnan Tumor Hospital, Kunming, China; ^4^ Departments of Combination of Traditional Chinese and Western Medicine, The Third Affiliated Hospital of Kunming Medical University/Yunnan Tumor Hospital, Kunming, China; ^5^ Department of Radiology, The Third Affiliated Hospital of Kunming Medical University/Yunnan Tumor Hospital, Kunming, China; ^6^ Department of Surgery Teaching Management, The Third Affiliated Hospital of Kunming Medical University/Yunnan Tumor Hospital, Kunming, China

**Keywords:** Hypoxia, immune, lncRNA, colorectal cancer, prognosis

## Abstract

**Background:**

Colorectal cancer (CRC) is one of the most common digestive system tumors worldwide. Hypoxia and immunity are closely related in CRC; however, the role of hypoxia-immune–related lncRNAs in CRC prognosis is unknown.

**Methods:**

Data used in the current study were sourced from the Gene Expression Omnibus and The Cancer Genome Atlas (TCGA) databases. CRC patients were divided into low- and high-hypoxia groups using the single-sample gene set enrichment analysis (ssGSEA) algorithm and into low- and high-immune groups using the Estimation of STromal and Immune cells in MAlignant Tumours using Expression data (ESTIMATE) algorithm. Differentially expressed lncRNAs (DElncRNAs) between low- and high-hypoxia groups, low- and high-immune groups, and tumor and control samples were identified using the limma package. Hypoxia-immune–related lncRNAs were obtained by intersecting these DElncRNAs. A hypoxia-immune–related lncRNA risk signature was developed using univariate Cox regression and least absolute shrinkage and selection operator (LASSO) analyses. The tumor microenvironments in the low- and high-risk groups were evaluated using ssGSEA, ESTIMATE, and the expression of immune checkpoints. The therapeutic response in the two groups was assessed using TIDE, IPS, and IC50. A ceRNA network based on signature lncRNAs was constructed. Finally, we used RT-qPCR to verify the expression of hypoxia-immune–related lncRNA signatures in normal and cancer tissues.

**Results:**

Using differential expression analysis, and univariate Cox and LASSO regression analyses, ZNF667-AS1, LINC01354, LINC00996, DANCR, CECR7, and LINC01116 were selected to construct a hypoxia-immune–related lncRNA signature. The performance of the risk signature in predicting CRC prognosis was validated in internal and external datasets, as evidenced by receiver operating characteristic curves. In addition, we observed significant differences in the tumor microenvironment and immunotherapy response between low- and high-risk groups and constructed a CECR7–miRNA–mRNA regulatory network in CRC. Furthermore, RT-qPCR results confirmed that the expression patterns of the six lncRNA signatures were consistent with those in TCGA-CRC cohort.

**Conclusion:**

Our study identified six hypoxia-immune–related lncRNAs for predicting CRC survival and sensitivity to immunotherapy. These findings may enrich our understanding of CRC and help improve CRC treatment. However, large-scale long-term follow-up studies are required for verification.

## Introduction

Colorectal cancer (CRC) is one of the most common digestive system tumors worldwide. With the widespread implementation of early CRC screening, the early diagnosis rate of CRC has improved significantly. However, its mortality rate remains relatively high. CRC is the second major cause of cancer-related deaths (1). Distant invasion and metastasis are the primary causes of death. Approximately 20% of CRC patients are newly diagnosed with distant metastasis, and an additional 25% of them will develop distant metastasis during treatment or follow-up (2). Currently, there are no effective biomarkers or prediction models for monitoring such patients in real time. Therefore, we still need to identify the key molecules involved in the development of CRC and develop new effective biomarkers and prognosis models to provide direction for the prevention, diagnosis, and treatment of CRC.

Oxygen homeostasis is essential for the survival of organisms. Hypoxia is the most common phenomenon in solid tumors and causes high oxygen consumption by cancer cells and abnormal blood supply in the tumor tissue (3). Extensive studies have demonstrated that hypoxia, mediated by hypoxia inducible factors (HIFs), can lead to a series of gene changes and changes in the tumor microenvironment and may also affect the proliferation, invasion, metastasis, and treatment resistance of cancer cells (4). Similar to other solid tumors, hypoxia has also been observed during CRC progression (5). More and more studies have demonstrated the key role of hypoxia in epithelial-to-mesenchymal transition (EMT), autophagy, 5-fluorouracil resistance, and poor prognosis of CRC, and have discussed the potential mechanism and treatment targets (6–8). For example, Hua et al. (9) found that, under hypoxic conditions, lncRNA LUCAT1 interacts with polypyrimidine-binding protein 1 (PTBP1) in CRC cells and promotes the combination of PTBP1 and DNA damage-related genes, which leads to a change in the variable splicing of these genes and promotes cell tolerance to DNA damage drugs (such as oxaliplatin), ultimately resulting in CRC cell proliferation. Recent studies have shown that hypoxia plays an important role in the regulation of the tumor immune microenvironment. Qi et al. (10) analyzed 1,730 CRC samples and found that hypoxia was closely related to higher M2 macrophage infiltration in CRC tissue, suggesting a poor prognosis in CRC patients. Malier et al. (11) found that hypoxia can lead to the specific overexpression of dihydropyrimidine dehydrogenase (DPD) in tumor-associated macrophages (TAMs), resulting in 5-FU resistance. Hypoxia and the HIF-1αsignaling pathway activated by photodynamic therapy (PDT) can lead to the upregulation of PD-L1, thus increasing the PD-L1 blocking therapy response in CRC (12). In view of the important role of hypoxia in the poor prognosis of CRC patients and immune microenvironment regulation, identifying the key regulatory factors between hypoxia and immunity is of important essential scientific significance, both for understanding the mechanisms of CRC progression and the choice of treatment strategies for CRC patients.

Long non-coding RNAs (lncRNAs) are a class of transcripts longer than 200 nucleotides. Studies have revealed that lncRNAs are involved in a series of cell biological processes, such as transcription regulation, mRNA post-transcriptional regulation, protein stability, subcellular structure, and epigenetic regulation, which contribute to the development of many types of tumors. LncRNAs are also considered potential markers for tumor diagnosis, prognosis, and treatment efficacy evaluation (13). He et al. (14) screened two lncRNAs, LINC01234 and MIR21HG, for prognosis evaluation in CRC patients using The Cancer Genome Atlas (TCGA) and clinical samples. Mo et al. (15) confirmed that lncRNA LDLRAD4-AS1 can destroy the stability of low-density lipoprotein receptor class A domain containing 4 (LDLRAD4) mRNA and reduce its expression, upregulate Snail expression, induce EMT, and promote the invasion and metastasis of CRC. Recently, many lncRNAs have been identified as being closely related to hypoxia. For example, Wang et al. (16) found that HIF-1α can increase the expression of lncRNA pituitary tumor-transforming 3, pseudogene (PTTG3P) by combining with the promoter region of PTTG3P and induce the differentiation and infiltration of M2 macrophages in CRC. Hypoxia-associated lncRNAs (HALs) are considered key factors in hypoxia-regulated tumor immunity (17).

The purpose of this study was to identify lncRNAs closely related to hypoxia and immunity through the public resources of CRC patients from TCGA and Gene Expression Omnibus (GEO) databases, construct a hypoxia-immune–related lncRNA signature, and explore the potential value of the signature in the prognosis and immunotherapy sensitivity prediction of CRC patients. Our research provides a theoretical basis for further research to explore the mechanism of hypoxia-immune–related lncRNAs in CRC, enrich our understanding of CRC, and help improve the treatment of CRC.

## Materials and methods

### Data source

**Figure 1:**
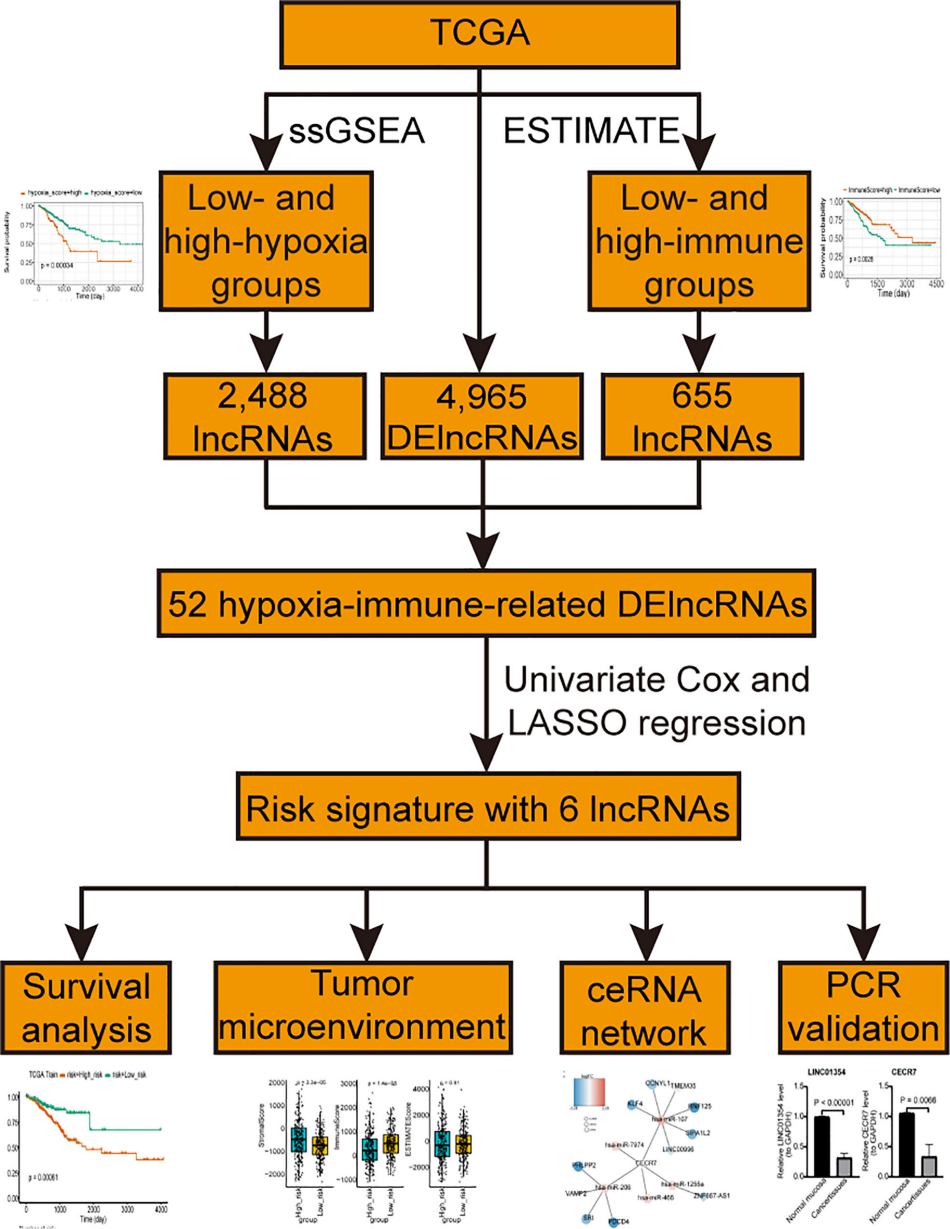
The flowchart of the steps involved in this study.

The lncRNA and mRNA expression profiles of 638 CRC and 51 control samples and the miRNA expression profiles of 615 CRC and 11 control samples were downloaded from TCGA database for differential expression analysis. Among them, 533 CRC samples with disease free survival (DFS) were used to screen prognostic lncRNAs, and 503 samples with complete clinical information were used to construct the nomogram. Additionally, 76 CRC patients with survival information in the GSE17538 dataset were sourced from the GEO database and used as the external validation dataset. In total, 510 hypoxia-related genes were extracted from the UniProt database (**Table S1**). The flowchart of the steps involved in this study is shown in **Figure 1**.

### Identification of hypoxia-immune–related DElncRNAs in CRC

Single-sample gene set enrichment analysis (ssGSEA) and ESTIMATE algorithms were used to evaluate hypoxia and immune enrichment scores, respectively. According to the optimal threshold calculated using the Survminer package, CRC patients were divided into low- and high-hypoxia groups or into low- and high-immune groups. Kaplan–Meier curves (K-M) and log-rank tests were performed to compare the CRC DFS between the low- and high-hypoxia or low- and high-immune groups. The functional enrichment of genes in the low- and high-hypoxia or low- and high-immune groups was performed using GSEA. DElncRNAs between low- and high-hypoxia groups, between low- and high-immune groups, and between CRC and control samples were identified using the limma R package with *p*< 0.05. The intersection of these DElncRNAs was defined as the hypoxia-immune–related DElncRNAs.

### Construction and validation of the risk signature

Patients from TCGA-CRC were divided into training (*n* = 374) and internal testing (*n* = 159) sets at a ratio of 7:3. Univariate Cox regression was first applied to obtain hypoxia-immune–related DElncRNAs that were significantly related to survival (*p*< 0.05). Subsequently, the least absolute shrinkage and selection operator (LASSO) algorithm was applied to identify the prognostic lncRNAs. We then determined the risk score using $\sum_{1}^{n}ExpGenei*Coefi.$ Based on the median risk score, patients with CRC were divided into low- and high-risk groups. The DFS of the low- and high-risk groups was evaluated using K-M curves. ROC curves were generated using the survival ROC package to assess the performance of the risk signature. Independent prognostic factors for CRC were screened using univariate and multivariate analyses. A nomogram was established to predict the 1-, 3-, and 5-year DFS rates of CRC patients. Calibration and decision curves were plotted to assess the clinical utility of the nomogram.

### Exploration of features in low- and high-risk groups

To characterize the low- and high-risk groups, we (i) compared the distribution of clinical features between the two groups using the χ² test; (ii) performed GSEA to explore biological functions enriched in the two groups; and (iii) compared the expressions of human leukocyte antigens (HLAs) and immune checkpoints, immunotherapy [TIDE (tumor immune dysfunction and exclusion) score and IPS (immunophenoscore)] and chemotherapy (IC50) response, and tumor microenvironment (immune/stromal/ESTIMATE score and immune cell infiltration) between the two groups using the Wilcoxon test.

### Construction of a prognosis-related ceRNA network in CRC

DEmiRNAs and DEmRNAs between tumor and control samples were screened using the limma R package with *p*< 0.05, |log_2_FC| > 0.5 and *p*< 0.05 and |log_2_FC| > 1 as criteria. miRanda software was used to predict DEmiRNAs interacting with prognostic lncRNAs, and starBase was used to identify DEmRNAs targeted by the predicted DEmiRNAs. Then lncRNA–miRNA–mRNA relationship was generated and viewed using Cytoscape.

### Validation of hypoxia-immune–related DElncRNAs by RT-qPCR

The total RNA from tumor (*n* = 10) and adjacent non-tumor (*n* = 10) samples from CRC patients was extracted using a TRIzol Reagent (Life Technology, CA, USA) according to the manufacturer’s instructions. After determining the concentration and purity of RNA, qualified RNA was used for reverse transcription using a SweScript RT I first-strand cDNA synthesis kit (Servicebio, Wuhan, China). qPCR was performed using 2 × Universal Blue SYBR Green qPCR Master Mix (Servicebio, Wuhan, China) under the following thermal cycling conditions: 40 cycles at 95°C for 60 s, 95°C for 20 s, 55°C for 20 s, and 72°C for 30 s. The qPCR results were further validated by 1% agarose gel electrophoresis experiments. The molecular weights of the amplification products of the six primers were consistent with the designed primers, and the product of each primer has no excess of spurious bands. The 2^-△△Ct^ method was used to calculate gene expressions. The primers used are listed in **Table 1**.

**Table 1:** Primers used in the current study.

Genes	Forward	Reverse
LINC01354	GAGAAGCGTGGGTAGGTATC	AGGAGGAAGTGGAAGTTGAA
CECR7	GGTCATCGCCATTCTCTAGT	GGCACAGTCAGGTCTTTCTC
ZNF667-AS1	TGTGCAGGATGATGCCACTTC	TGTTACTCCCCAGACCGAGAG
LINC01116	AGGCCCTGAAGTACACAGTTTTCT	CTTTGCCATTCTGATGTTACCCAC
LINC00996	CCCACCAAACCCAAAACAAAC	TCCAACTACTCGGGAGACAGC
DANCR	ACTATGTAGCGGGTTTCGG	CTGCTCTAGCTCCTGTGGC
GAPDH	GGAAGGTGAAGGTCGGAGT	TGAGGTCAATGAAGGGGTC

## Results

### CRC patients were divided into low- and high-hypoxia groups

**Figure 2:**
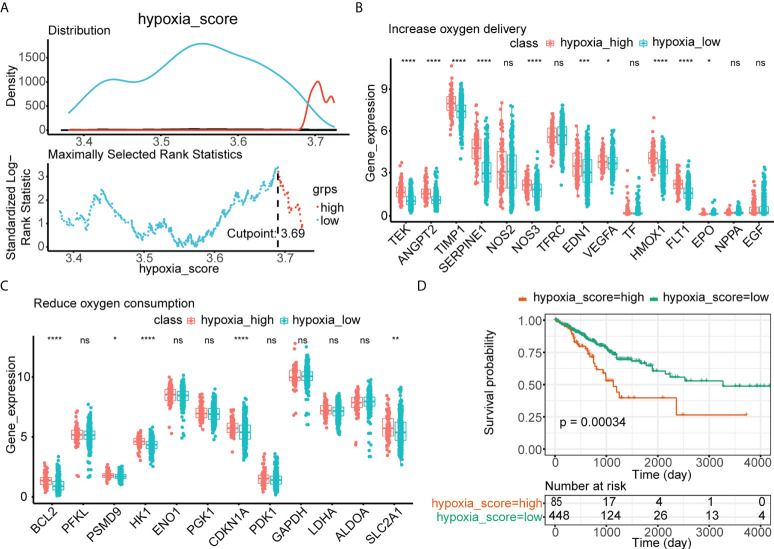
Identification of low- and high-hypoxia groups in TCGA. **(A)** The optimal threshold of the hypoxia enrichment score calculated by ssGSEA. **(B)** The expressions of 15 increased oxygen delivery genes in hypoxia-high and hypoxia-low groups in TCGA. **(C)** The expressions of 12 reduced oxygen consumption genes in hypoxia-high and hypoxia-low groups in TCGA. **(D)** Kaplan–Meier analysis of TCGA-CRC patients’ survival in low- and high-hypoxia groups. ns, no significance; *p< 0.05; **p< 0.01; ***p< 0.001; ****p< 0.0001.

Based on the optimal threshold of the hypoxia enrichment score calculated by ssGSEA, CRC patients from TCGA were divided into low- and high-hypoxia groups (**Figure 2A**). Patients in the high-hypoxia group had higher expressions of genes related to increased oxygen delivery (**Figure 2B**) and reduced oxygen consumption (**Figure 2C**) but worse DFS than those in the low-hypoxia group (**Figure 2D**). GSEA showed that immune and extracellular matrix (ECM)–related Kyoto Encylopaedia of Genes and Genomes (KEGG) pathways and Gene Ontology (GO) terms were significantly enriched in the high-hypoxia group, such as KEGG pathways of cytokine–cytokine receptor interaction, focal adhesion, and ECM receptor interaction (**Supplementary Figure 1A**) and GO terms of biological adhesion, defense response, and cell migration (**Supplementary Figure 1B**). A total of 2,488 DElncRNAs were found between the low- and high-hypoxia groups (**Supplementary Figure 1C**), the expressions of which are displayed in the heatmap (**Supplementary Figure 1D**). CRC patients from TCGA cohort were divided into two hypoxia subgroups with significant survival differences and immune and ECM-related KEGG pathway variances.

### CRC patients were divided into low- and high-immune groups

**Figure 3:**
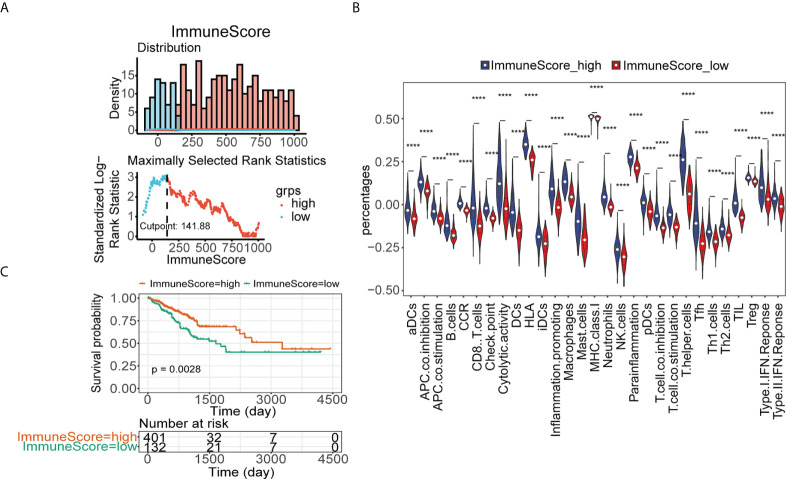
Identification of low- and high-immune groups in TCGA. **(A)** The optimal threshold of immune score calculated by ESTIMATE. **(B)** The abundance of 29 immune cells estimated by ssGSEA in immune score high and immune score low groups in TCGA. **(C)** Kaplan–Meier analysis of TCGA-CRC patients’ survival in low- and high-immune score groups ****p< 0.0001.

Similarly, based on the optimal threshold of the immune enrichment score calculated by ESTIMATE, CRC patients from TCGA were divided into low- and high-immune groups (**Figure 3A**). Patients in the high-immunity group had higher infiltration of immune cells (**Figure 3B**) and better survival than those in the low-immunity group (**Figure 3C**). GSEA showed that immune-related KEGG pathways and GO terms were significantly enriched in the high-immune group, such as the KEGG pathways of antigen processing and presentation, chemokine signaling pathway, cytokine–cytokine receptor interaction (**Supplementary Figure 2A**), and GO terms of the cytokine-mediated signaling pathway, defense response, and immune effector response (**Supplementary Figure 2B**). Thereafter, 655 DElncRNAs were found between the low- and high-immune groups (**Supplementary Figure 2C**), and their expression is shown in the heatmap (**Supplementary Figure 2D**). CRC patients from TCGA cohort were divided into two immune subgroups with significant survival differences and immune-related KEGG pathway variances.

### Identification of 52 hypoxia-immune–related DElncRNAs in CRC

**Figure 4:**
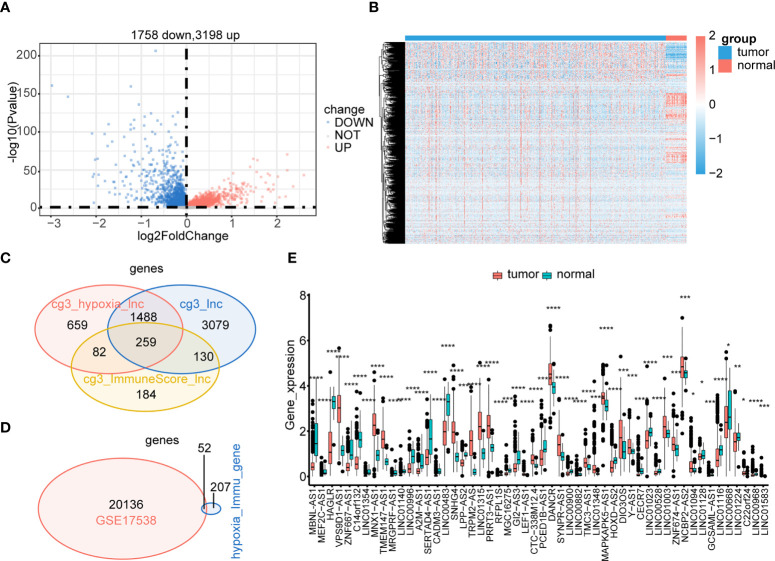
Identification of hypoxia-immune–related DElncRNAs. Expressions of tumor-related lncRNAs are displayed in the volcano plot **(A)** and heatmap **(B)**. Hypoxia-immune–related DElncRNAs were identified in the hypoxia-related lncRNAs group, immune-related lncRNAs group, and tumor-related lncRNAs group of TCGA **(C)**. Common hypoxia-immune–related DElncRNAs were identified in TCGA and GSE17538 **(D)**. The expression levels of 52 common hypoxia-immune–related DElncRNAs in TCGA **(E)**. ****p< 0.0001.

To identify the hypoxia-immune–related DElncRNAs involved in CRC, we also screened 4,965 DElncRNAs between the tumor and control samples (**Figures 4A, B**). By intersecting DElncRNAs between the low- and high-hypoxia groups, between low- and high-immune groups, and tumor and control samples in TCGA, we obtained 259 DElncRNAs in TCGA (**Figure 4C**). These DElncRNAs further overlapped with lncRNAs in GSE17538, and 52 hypoxia-immune–related DElncRNAs were selected for downstream analyses (**Figure 4D**). The expression levels of 52 hypoxia-immune–related DElncRNAs were significantly different between the tumor and control samples from TCGA (**Figure 4E**).

### A hypoxia-immune–related risk signature was developed in CRC

**Figure 5:**
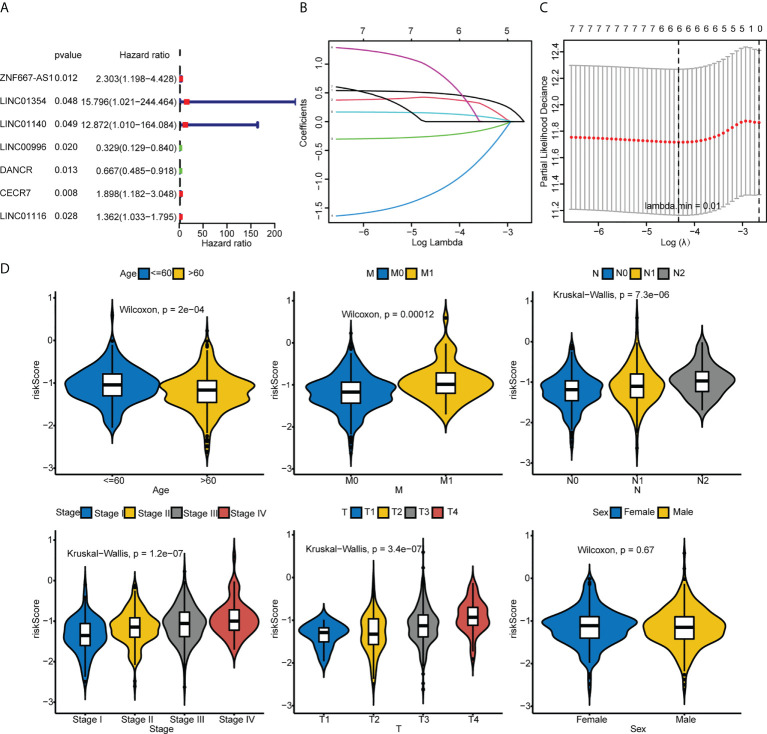
Identification of the hypoxia-immune–related risk signature. **(A)** Univariate Cox regression analysis of 52 common hypoxia-immune–related DElncRNAs in TCGA. **(B, C)** LASSO analysis was performed to identify the prognostic signature. **(D)** Comparison of the risk scores between or among groups stratified by different clinical features.

**Figure 6:**
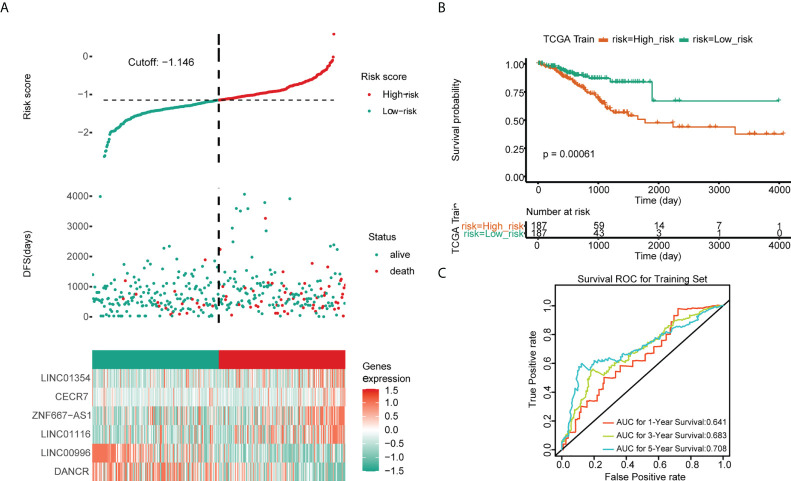
Predictive values of the risk signature model in TCGA training set. **(A)** The risk score and survival status plots. **(B)** Kaplan–Meier analysis of patients’ survival in the low- and high-risk groups. **(C)** ROC curves of the risk score model in predicting the 1-, 3-, and 5-year DFS of CRC patients.

**Figure 7:**
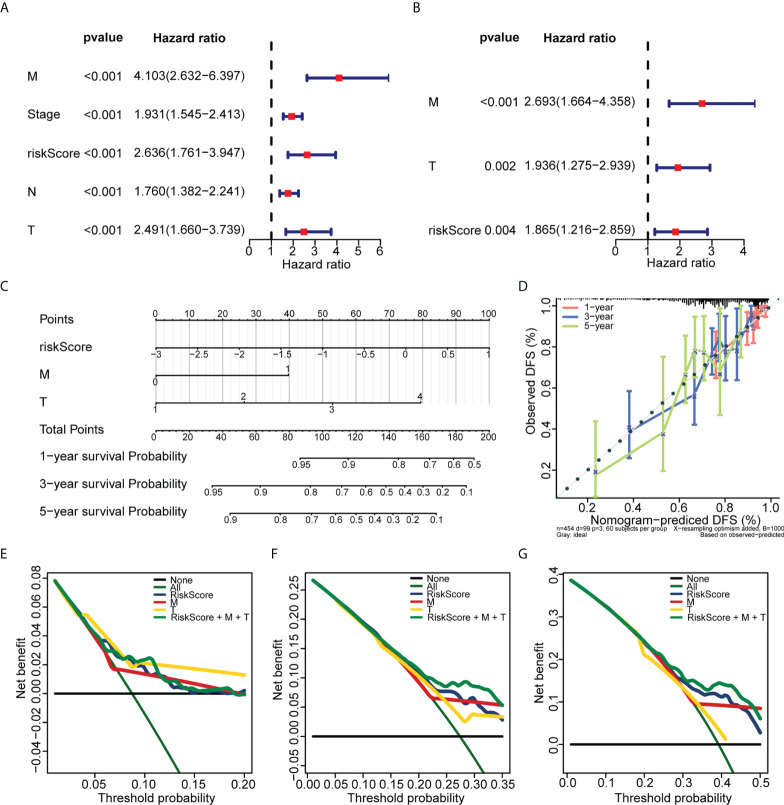
Establishment of the nomogram used for predicting the prognosis of CRC patients. **(A)** Univariate Cox regression analysis of the risk score and clinical variables of CRC patients in TCGA. **(B)** Multivariate Cox regression analysis of the risk score and clinical variables of CRC patients in TCGA. **(C)** The nomogram was constructed based on T, M, and risk score. **(D)** The calibration plots of the nomogram for predicting the 1-, 3-, and 5-year overall survival. (E, F, and G) The decision curves for 1 **(E)**, 3 **(F)**, and 5 years **(G)**.

Univariate Cox regression analysis revealed that ZNF667-AS1, LINC01354, LINC01140, LINC00996, DANCR, CECR7, and LINC01116 were significantly associated with prognosis (*p*< 0.05, **Figure 5A**). LASSO was then performed, and ZNF667-AS1, LINC01354, LINC00996, DANCR, CECR7, and LINC01116 were selected as prognostic signatures (**Figures 5B, C**) to construct the risk signature. We found that the risk signature was associated with multiple clinical features of CRC, including age, tumor stage, and TNM staging (**Figure 5D**). The risk score of each sample from the training, test, and validation sets was calculated using the formula: Risk score = 0.4856 × expCECR7 + (−0.2392) × expDANCR + (−1.15) × expLINC00996 + 0.04 × expZNF667-AS1 + 0.1411 × expLINC01116 + 0.7806 × expLINC01354, and the samples from the three datasets were divided into high- and low-risk groups according to the median risk score. Patients in TCGA training set were assigned to low- and high-risk groups with markedly different DFS according to the median risk score (**Figures 6A, B**). ROC curves revealed that the risk signature could predict CRC prognosis with areas under the curve (AUCs) greater than 0.6 (**Figure 6C**). Consistent results were obtained in TCGA testing set (**Supplementary Figures 3A–C**) and GSE17538 datasets (**Supplementary Figures 3D–F**). Next, using univariate and multivariate analyses, we found that T and M staging and risk score may be independent prognostic factors for CRC (**Figures 7A, B**). Based on these results, a nomogram was constructed (**Figure 7C**), the calibration curves of which showed that the predicted survival at 1, 3, and 5 years was similar to the actual survival (**Figure 7D**). The clinical characteristics of different risk groups in the training and test sets from TCGA are shown in **Supplementary Table 2** (**Table S2**). Moreover, the decision curves revealed that the nomogram had more benefits than treat-all, treat-none, risk score, T staging, and M staging alone at 3 and 5 years (**Figures 7E-G**). These results indicated that the constructed risk model based on the expression of hypoxia-immune–related lncRNAs was associated with the clinical characteristics of CRC and could predict the survival time of CRC patients, and that these lncRNAs are prognostic biomarkers of CRC. Furthermore, the risk score is a potent marker for predicting the prognosis of patients with CRC.

### Different characteristics were observed between the high- and low-risk groups

**Figure 8:**
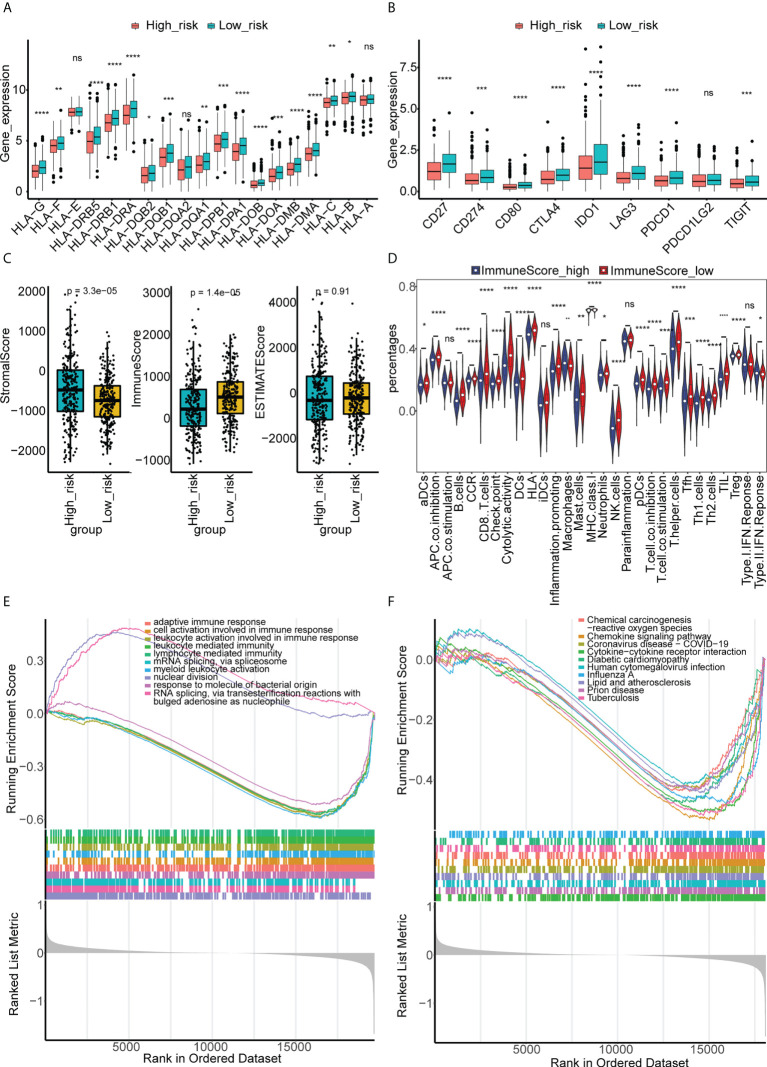
Different immune characteristics and GSEA enrichment between the high- and low-risk groups. **(A)** The expressions of HLA genes in the high- and low-risk groups of CRC in TCGA. **(B)** The expressions of immune checkpoints in the high- and low-risk groups of CRC in TCGA. **(C)** Immune, stromal, and ESTIMATE scores were compared between high- and low-risk groups of CRC in TCGA. **(D)** The abundance of 29 immune cells estimated by ssGSEA in the high- and low-risk groups of CRC in TCGA. **(E)** GSEA of differentially expressed genes between the high- and low-risk groups of CRC in GO terms. **(F)** GSEA of differentially expressed genes between the high- and low-risk groups of CRC in KEGG terms. "ns, no significance; *p< 0.05, **p< 0.01, ***p< 0.001, and ****p< 0.0001.

We found that there were more CRC patients at M1, N2, stage IV, and T4 in the high-risk group (**Table 1**), indicating that the risk signature was related to CRC progression. The expression of HLAs (**Figure 8A**), immune checkpoints (**Figure 8B**), immune/stromal/ESTIMATE scores (**Figure 8C**), and immune cell infiltration (**Figure 8D**) were much higher in the low-risk group. GSEA also showed that immune-related GO terms and KEGG pathways, such as adaptive immune response, chemokine signaling pathway, and leukocyte-mediated immunity, were significantly enriched in the low-risk group (**Figures 8E, F**). These results suggest that the two groups had different tumor microenvironments. A close relationship between the tumor microenvironment and therapeutic responses has been widely reported (18–20). Therefore, we compared the therapeutic response between the two groups. We found that patients in the low-risk group had a lower TIDE (**Supplementary Figure 4A**) and higher IPS scores (**Supplementary Figure 4B**), indicating that they had a better response to immunotherapy. In addition, significant differences of sensitivity to 33 drugs were found between the two groups (**Table S3**). Herein, we identified 18 drugs with significant IC50 values between the two groups (**Supplementary Figure 5**).

### The ceRNA network based on prognostic lncRNAs was constructed in CRC

**Figure 9:**
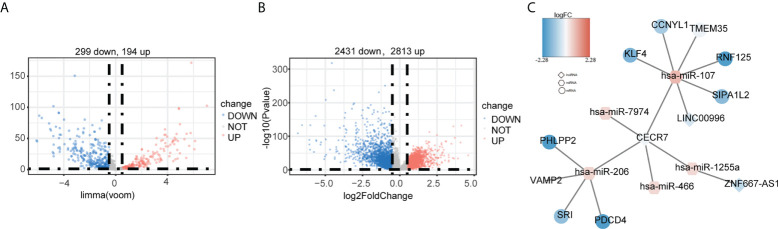
Construction of the ceRNA network based on the six hypoxia-immune–related lncRNAs. **(A)** Identification of differentially expressed miRNAs between cancer and normal tissues in TCGA. **(B)** Identification of differentially expressed mRNAs between cancer and normal tissues in TCGA. **(C)** ceRNA network. Round: mRNA; hexagon: miRNA; diamond: lncRNA.

Finally, we constructed a ceRNA network for CRC. First, 493 DEmiRNAs (**Figure 9A**) and 5,219 DEmRNAs (**Figure 9B**) were identified between tumor and control samples. Using miRanda and starBase software, we found that the prognostic hypoxia-immune–related lncRNA, CECR7, may interact with mir-206 and mir-107 to regulate the expression of the PH domain and leucine-rich repeat protein phosphatase 2 (PHLPP2), vesicle associated membrane protein 2 (VAMP2), sorcin (SRI), programmed cell death 4 (PDCD4), Kruppel-like factor 4 (KLF4), cyclin Y like 1 (CCNYL1), transmembrane protein 35 (TMEM35), ring finger protein 125 (RNF125), and signal-induced proliferation associated 1 like 2 (SIPA1L2) (**Figure 9C**).

### The expressions of signature lncRNAs *in vivo*

**Figure 10:**
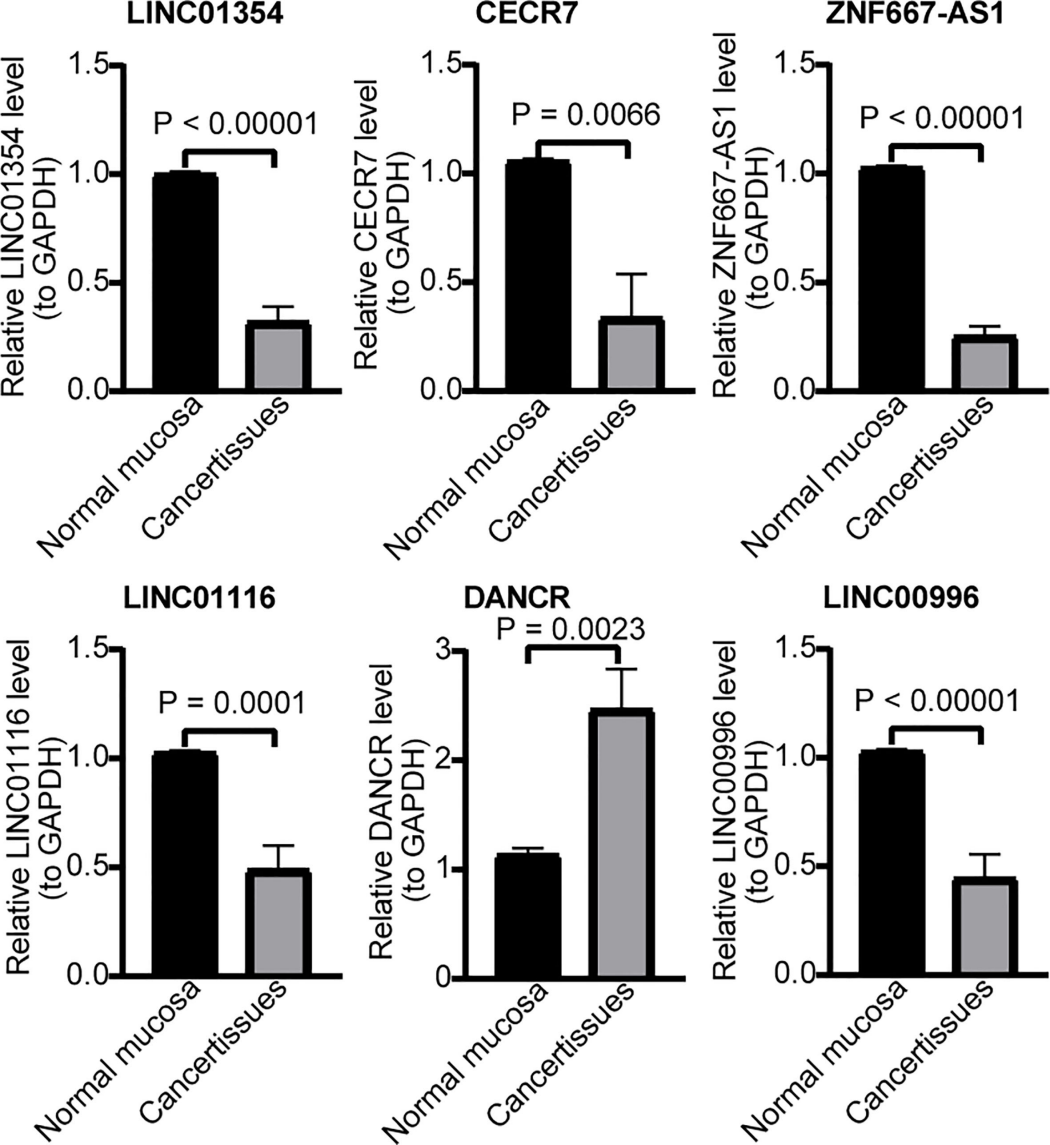
RT-qPCR validation for hypoxia-immune–related genes. Expression of LINC01354, CECR7, ZNF667-AS1, LINC01116, DANCR, and LINC00996 in CRC and normal mucosa tissues.

Finally, we examined their expression by RT-qPCR and found that the expression patterns of the six lncRNA signatures were completely consistent with those in TCGA-CRC cohort. Specifically, CRC samples had a lower expression of ZNF667-AS1, LINC01354, LINC01140, LINC00996, CECR7, and LINC01116 and a higher expression of DANCR than the controls (**Figure 10**).

## Discussion

Hypoxia and immune escape are two major hallmarks of cancer (21). Studies have shown that hypoxia plays a key role in regulating the immune microenvironment and promoting immune escape in CRC (22). lncRNAs have also been shown to be key regulators of the occurrence and development of CRC (23). However, the role of hypoxia-immune–related lncRNAs in CRC has not been completely elucidated. In this study, six hypoxia-immune–related lncRNAs were identified from TCGA and GEO public databases of CRC, including ZNF667-AS1, LINC01354, LINC00996, DANCR, CECR7, and LINC01116. A hypoxia-immune–related lncRNA signature was constructed using the identified lncRNAs. The signature presented good application value in prognosis prediction and the immunotherapy response of CRC patients. These results were verified using both internal and external datasets. Later, we divided the patients into low- and high-risk groups based on the signature and found that there were significant differences in the tumor microenvironment and immunotherapy response between the two groups.

We analyzed the KEGG pathways and GO term enrichment of differentially expressed genes in patients between the high- and low-hypoxia groups. Immunity- and extracellular matrix–related KEGG pathways and GO terms of biological adhesion, defense response, and cell migration were significantly enriched in the high-hypoxia group. Under hypoxia, extracellular matrix remodeling-related pathways, such as focal adhesion and ECM receptor interaction, are important mechanisms for distant metastasis of CRC (24, 25). Immune-related pathways, such as cytokine–cytokine receptor interactions, are also abnormally activated in tumors under hypoxia (26). Moreover, studies on CRC have shown that cytokine–cytokine receptor interaction is involved in changes in the tumor microenvironment and has a good guidance value in the prognosis and immunotherapy prediction of CRC patients (27, 28). KEGG pathways and GO term enrichment of differentially expressed genes in patients between the high- and low-immune groups were also analyzed. The results also suggested that the immune-related KEGG pathways and GO terms of the cytokine-mediated signaling pathway, defense response, and immune effector response were significantly enriched in patients with high immunity. Six hypoxia-immune–related lncRNAs, ZNF667-AS1, LINC01354, LINC00996, DANCR, CECR7, and LINC01116, were identified for the construction of signatures in CRC patients. The patients were divided into high- and low-risk groups based on this signature. The immune-related KEGG pathways and GO terms, such as adaptive immune response, chemokine signaling pathway, and leukocyte-mediated immunity, were significantly enriched in the low-risk group. Therefore, immune-related pathways may be important mechanisms for hypoxia-immune related lncRNAs in the regulation of the tumor immune microenvironment and progression of CRC.

Studies have shown that four hypoxia-immune–related lncRNAs, DANCR, ZNF667-AS1, LINC01354, and LINC01116, are involved in CRC development through different mechanisms. The role of DANCR in CRC has been intensively studied; DANCR can induce EMT and promote invasion and metastasis of HT29 cells (29). DANCR can also promote the metastasis of CRC cells through the DANCR/microRNA-518a-3p/MDMA ceRNA network (30) and DANCR/miR-185-5p/HMGA2 axis (31), inhibit KAT6A acetylation by interacting with lysine acetyltransferase 6A (32), and inhibit apoptosis by enhancing the mRNA stability of MALAT1 mRNA in CRC cells (33). The high expression of DANCR in patients with CRC was significantly related to the poor TNM stage. The detection of DANCR was more sensitive than that of CEA and CA199 in blood samples for the early diagnosis of CRC. DANCR is a potential biomarker for the early diagnosis of CRC (34). Zhuang et al. (35) suggested that ZNF667-AS1 can inhibit CRC progression by activating ANK2/JAK2 signaling. Li et al. (36) found that LINC01354 interacts with heterogeneous ribonucleoprotein D (hnRNPD) to promote the invasion and metastasis of CRC through the Wnt/β-catenin pathway. LINC01116 can inhibit TPM1 transcription by increasing EZH2 to enhance the methylation of T, the PM1 promoter region, promote the proliferation and angiogenesis of CRC (37), or negatively regulate miR-9-5p to induce STMN1 expression, inhibit apoptosis, and promote the invasion and metastasis of CRC cells (38). Only one report of bioinformatics analysis suggested that the expression of LINC00996 in CRC tissues is lower than that in normal tissues, and its low expression is related to the occurrence, metastasis, and poor prognosis of patients with CRC (39). At present, there are no relevant reports on CECR7 in CRC. This evidence supports our conclusion, and the results of the clinical samples verified the expression differences found by the bioinformatics analysis.

To explore the potential mechanism of the six lncRNAs in guiding the survival and prognosis of CRC, we constructed a risk-scoring system based on the six lncRNAs and divided the patients into high- and low-risk groups according to the system. Tumor immune microenvironment analysis showed that patients in the low-risk group had higher expression of multiple HLA and immune checkpoints, lower matrix score, higher immune score, and higher immune cell infiltration than patients in the high-risk group. KEGG pathway and GO term enrichment analysis showed that the adaptive immune response, chemokine signaling pathway, and leukocyte-mediated immunity were significantly enriched in low-risk patients. The interaction between tumor cells and the tumor immune microenvironment plays an important role in the development of tumor; however, the underlying mechanism is complex. Studies have shown that loss of HLA expression promotes tumor cell immune escape by weakening the ability of new antigen presentation (40, 41). However, some studies have suggested that HLA-G can be recognized as a potential immune checkpoint that promotes tumor immune escape (42). The high expression of immune checkpoints has been proven to be a key factor in inducing immune escape. As a negative regulator of T-cell activation, it also regulates the immune response to avoid T-cell over-activation. On the one hand, high expression of immune checkpoints promotes immune escape; on the other hand, immune checkpoints provide a potential target for tumor therapy. Blocking the immune checkpoint pathway favors antitumor T-cell responses (43, 44). CRC patients with high-immune scores, although with poor prognosis, easily benefit from adjuvant chemotherapy and immunotherapy (45). The composition of the immune cell is complex. Although the infiltration of immune cells has been proven to promote the development of tumors, increased immune cell infiltration can also create conditions for immunotherapy (46, 47). Therefore, the balance between immunosuppressive factors and immune activating factors in the tumor immune microenvironment is key to promoting or inhibiting cancer, and changes in the tumor immune microenvironment are also important factors affecting the efficacy of immunotherapy (48). Based on our results, higher expression of HLAs suggests that patients in the low-risk group had strong antigen presentation ability, higher expression of immune checkpoints, and higher immune scores and immune cell infiltration, suggesting that patients may benefit from immunotherapy.

Prediction of tumor drug sensitivity is of great significance for guiding clinical medication. The TIDE and IPS are important tools for predicting the effect of immunotherapy in patients with tumor. Studies have shown that lower TIDE and higher IPS suggest that patients with tumor can benefit significantly from immunotherapy; therefore, TIDE and IPS have been widely used to evaluate the effect of immunotherapy (49–51). Our results suggest that patients in the low-risk group have lower TIDE and higher IPS score; therefore, patients in the low-risk group were more sensitive to immunotherapy. IC50 is an important index for predicting chemosensitivity (52). Based on the IC50 results, we found significant differences in the sensitivity of patients in the high- and low-risk groups to 33 common chemotherapy drugs. Previous evidence suggests that LINC01116 can cause lung cancer to become resistant to cisplatin and gefitinib (53, 54). DANCR expression is an important factor leading to drug resistance in colon cancer, gastric cancer, and other tumors (55–57). LncRNA-DANCR-miR-125b-5p/HK2 is the key mechanism that mediates cisplatin resistance in colon cancer (57). Cisplatin is a first-generation platinum anticancer drug and is a traditional regimen for CRC. Our model had a good predictive effect on the sensitivity of CRC to cisplatin. However, our results do not suggest that this model can better predict the sensitivity of patients to classic chemotherapeutic drugs, such as oxaliplatin, fluorouracil, and irinotecan for CRC. However, the results have a guiding role in the selection of chemotherapy drugs for some patients with CRC. In conclusion, the risk signature established in this study can better predict the sensitivity of CRC patients to immunotherapy and has a reference value for the choice of immunotherapy for CRC patients.

The ceRNA network is a potential regulatory molecular mechanism mediated by lncRNAs and can provide indirect evidence for the exploration of biomarkers and therapeutic targets and the speculation of molecular mechanisms (58). Therefore, we constructed a hypoxia-immune–related lncRNAs–miRNA–mRNA ceRNA network. These results suggest that CECR7 plays a key role in CRC by regulating miR-206 and miR-107. Several studies have preliminarily explored the role of miR-206 and miR-107 in tumor progression and immunity. For example, in breast cancer, miR-206 is an effector of KLF4-mediated anoikis resistance and tumor development through repression of the PDCD4 (59). MiR107 promotes the invasion and metastasis of CRC by inhibiting KLF4 (60). Recent evidence shows that miR206 can promote the expression of CCL2 by inhibiting KLF4 in M1 macrophages, a subtype of Kupffer cells, to increase the level of CCR2^+^ cytotoxic T cells, thereby inhibiting the occurrence and development of HCC (61). Therefore, we speculated that CECR7 may play an important role in the development of CRC and in the regulation of the CRC immune microenvironment by regulating miR206 and miR107. However, the relationship between CECR7 and miR206/miR107 requires further verification.

In this study, a signature based on six hypoxia-immunity–related lncRNAs was established, and its value in survival prediction was verified using TCGA internal validation set and GEO external validation set. The potential application value of this signature in tumor immune microenvironment evaluation and immunotherapy efficacy prediction was evaluated. Our report is based primarily on a comprehensive bioinformatics approach. Although we simply verified the expression differences of the six lncRNAs in cancer tissues and normal tissues, the accuracy of these lncRNAs in predicting the prognosis, immune regulation, and immunotherapy efficacy of CRC patients still needs to be verified by large-sample cohort studies. At the same time, the role and mechanism of single lncRNAs or multi-lncRNA cooperation in CRC are worth exploring.

In conclusion, this study identified six hypoxia-immunity–related lncRNAs in CRC for the first time, explored their potential molecular mechanisms, and established a signature that can predict the prognosis and therapeutic response of CRC patients. Our results may provide a personalized prediction tool for the prognosis and efficacy of immunotherapy in CRC patients.

## Data availability statement

The datasets presented in this study can be found in online repositories. The names of the repository/repositories and accession number(s) can be found below: TCGA AND GEO. The accession number(s) can be found in the article/**Supplementary Material**.

## Ethics statement

This study was reviewed and approved by The ethics committee of Yunnan Cancer Hospital. The patients/participants provided their written informed consent to participate in this study.

## Author contributions

LL, XC and ZY contributed to the conception and design of the study. YD, TS, SL and ZL contributed to the writing, review, and/or revision of the manuscript. CY, ZC, SF and HQ provided administrative, technical, or material support. All authors contributed to the article and approved the submitted version.

## Funding

This study was supported by National Natural Science Foundation of China (82060516), Yunnan Fundamental Research Projects (202101AY070001-167)(202201AY070001-134)(2018FA040), “Famous Doctor” Fund Project of Yunnan Province (2021) and Innovation Fund for Postgraduates of Kunming Medical University (2022S314).

## Conflict of Interest

The authors declare that the research was conducted in the absence of any commercial or financial relationships that could be construed as a potential conflict of interest.

## Publisher’s note

All claims expressed in this article are solely those of the authors and do not necessarily represent those of their affiliated organizations, or those of the publisher, the editors and the reviewers. Any product that may be evaluated in this article, or claim that may be made by its manufacturer, is not guaranteed or endorsed by the publisher.

## Supplementary material

Supplementary Figure 1 Identification of biological function and hypoxia-related lncRNAs in TCGA. KEGG **(A)** and GO **(B)** analysis between hypoxia-high and hypoxia-low groups were performed by Gene set enrichment analysis (GSEA). Different expression of hypoxia-related lncRNAs was identified by volcano plot **(C)** and heatmap **(D)**.

Supplementary Figure 2 Identification of biological function and immune-related lncRNAs in TCGA. KEGG **(A)** and GO **(B)** analyses between high-immune score and low-immune score groups were performed by Gene set enrichment analysis (GSEA). Different expression of immune-related lncRNAs were identified by volcano plot **(C)** and heatmap **(D)**.

Supplementary Figure 3 Predictive values of the risk signature model in TCGA testing set and GSE17538 dataset. **(A)**The risk score and survival status plots. **(B)** Kaplan–Meier analysis of the survival of TCGA-CRC patients in the low- and high-risk groups. **(C)** ROC curves of the risk score model in predicting the 1-, 3-, and 5-year DFS of TCGA-CRC patients. **(D)** The risk score and survival status plots. **(E)** Kaplan–Meier analysis of the risk score model in predicting 1-, 3-, and 5-year DFS of GSE17538-CRC patients. **(F)** ROC curves of the risk score model in predicting the 1-, 3-, and 5-year DFS of TCGA- GSE17538-CRC patients.

Supplementary Figure 4 Immune therapeutic response prediction between the high- and low-risk groups. **(A)** TIDE prediction in the high- and low-risk groups of CRC in TCGA. **(B)** IPS prediction in the high- and low-risk groups of CRC in TCGA.

Supplementary Figure 5 Therapeutic response prediction of 33 drugs between the high- and low-risk groups.
