# Supplementary figures and images for: Development of a novel hypoxia-immune–related LncRNA risk signature for predicting the prognosis and immunotherapy response of colorectal cancer
Source: Front Immunol. 2022 Sep 14;13:951455. doi: 10.3389/fimmu.2022.951455 (PMC9516397; doi:10.3389/fimmu.2022.951455)

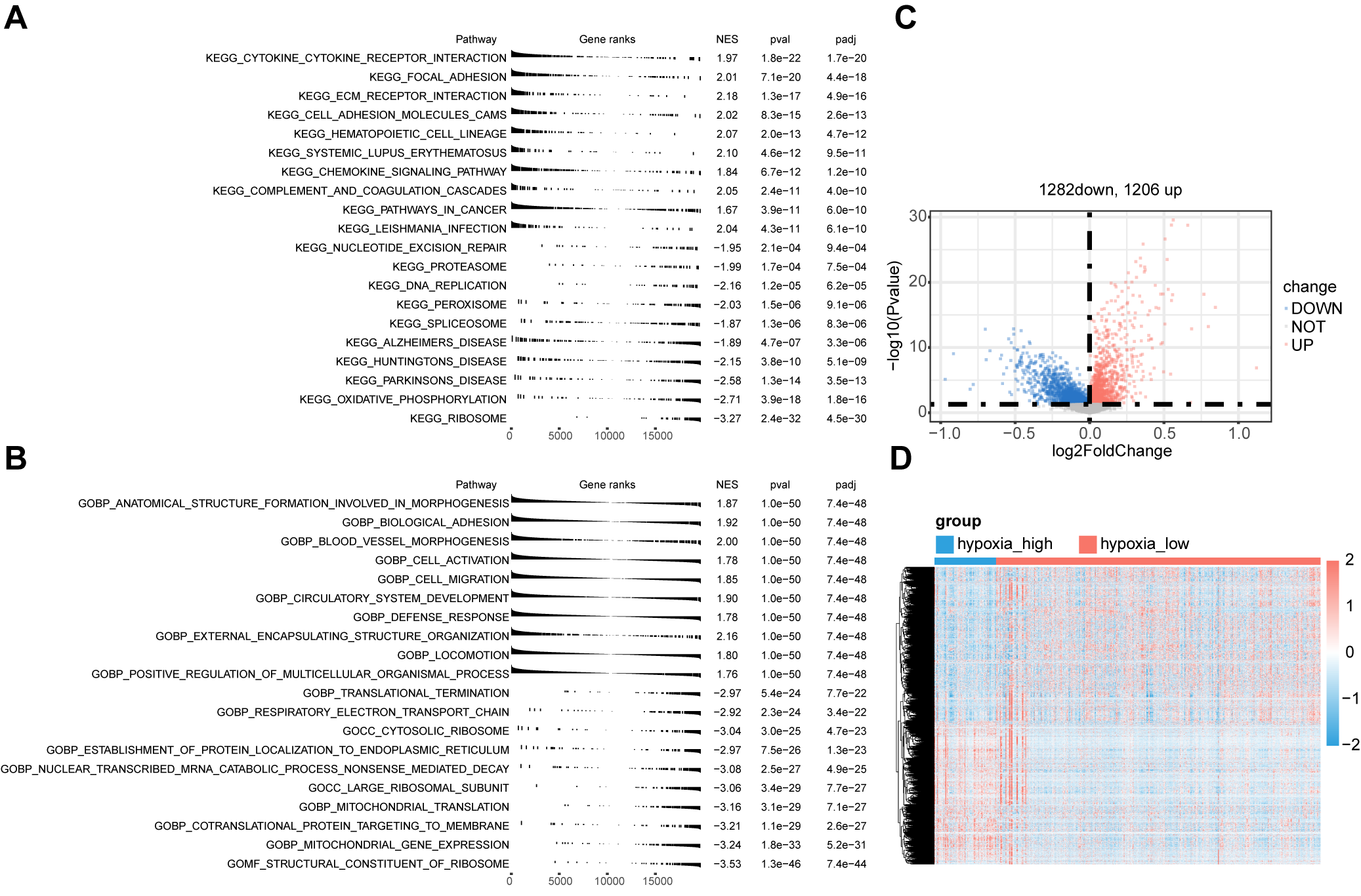

Supplement: Supplementary Figure 1 — Identification of biological function and hypoxia-related lncRNAs in TCGA. KEGG (A) and GO (B) analysis between hypoxia-high and hypoxia-low groups were performed by Gene set enrichment analysis (GSEA). Different expression of hypoxia-related lncRNAs was identified by volcano plot (C) and heatmap (D). [file Image_1.tif]

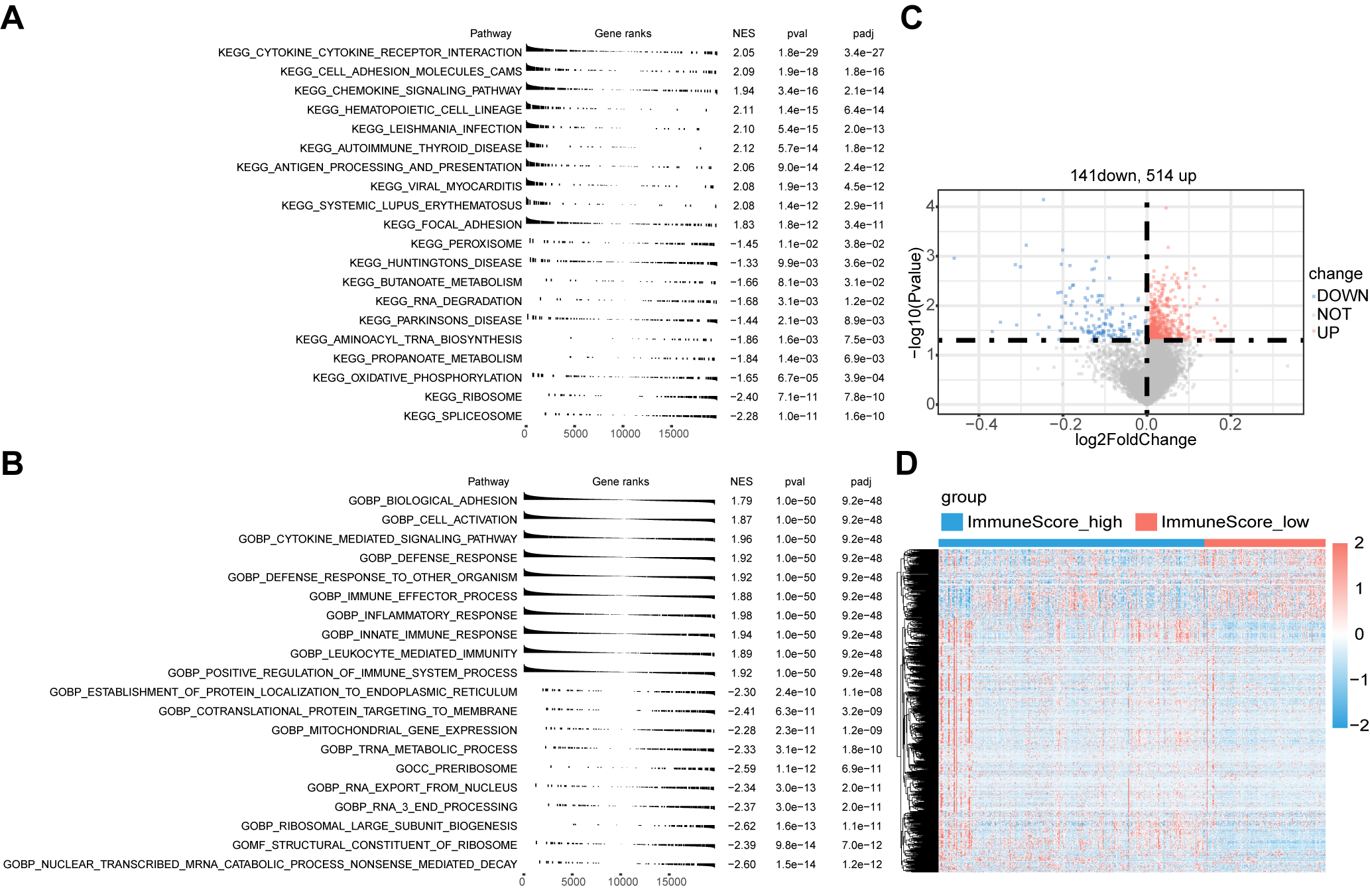

Supplement: Supplementary Figure 2 — Identification of biological function and immune-related lncRNAs in TCGA. KEGG (A) and GO (B) analyses between high-immune score and low-immune score groups were performed by Gene set enrichment analysis (GSEA). Different expression of immune-related lncRNAs were identified by volcano plot (C) and heatmap (D). [file Image_2.tif]

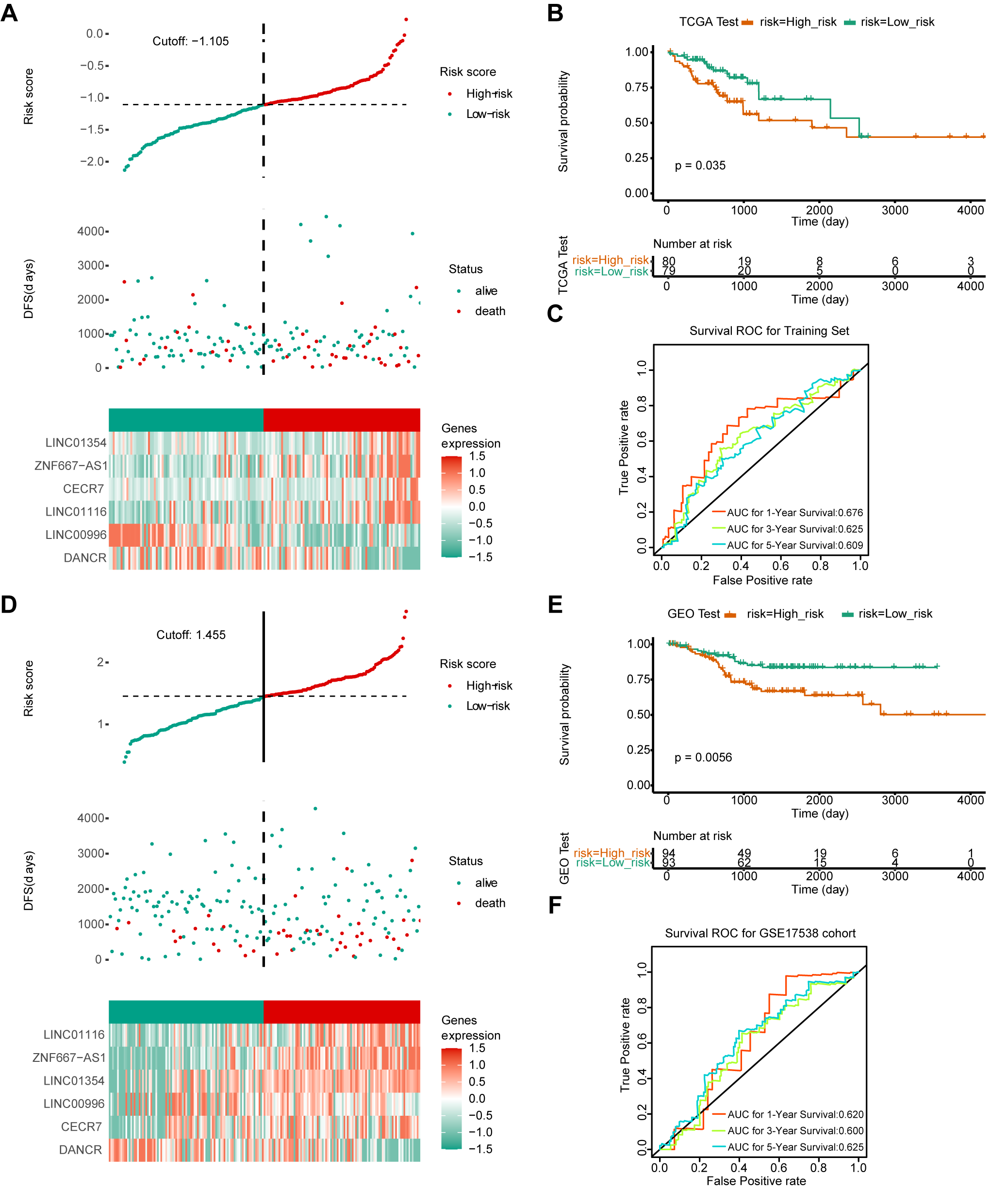

Supplement: Supplementary Figure 3 — Predictive values of the risk signature model in TCGA testing set and GSE17538 dataset. (A)The risk score and survival status plots. (B) Kaplan–Meier analysis of the survival of TCGA-CRC patients in the low- and high-risk groups. (C) ROC curves of the risk score model in predicting the 1-, 3-, and 5-year DFS of TCGA-CRC patients. (D) The risk score and survival status plots. (E) Kaplan–Meier analysis of the risk score model in predicting 1-, 3-, and 5-year DFS of GSE17538-CRC patients. (F) ROC curves of the risk score model in predicting the 1-, 3-, and 5-year DFS of TCGA- GSE17538-CRC patients. [file Image_3.tif]

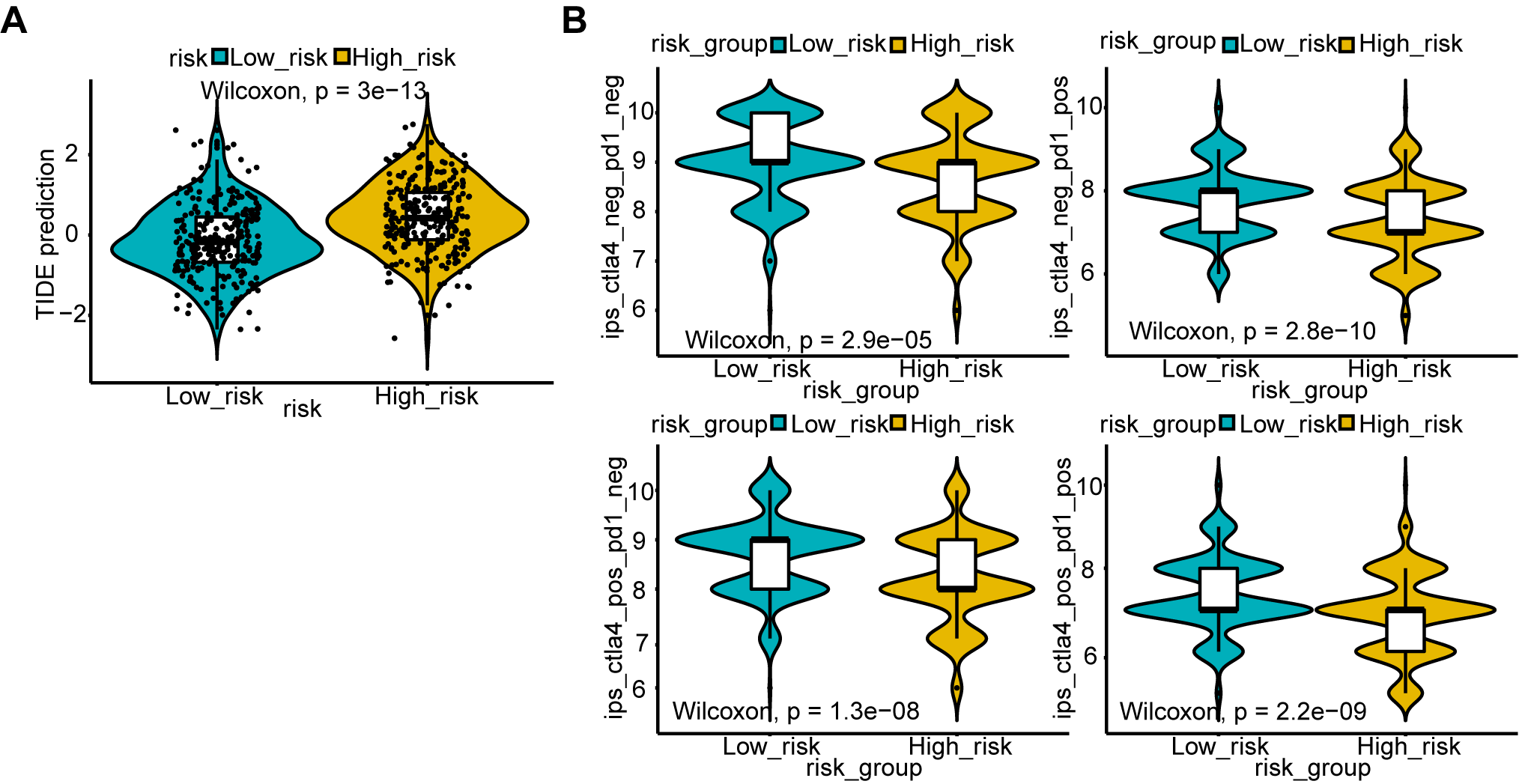

Supplement: Supplementary Figure 4 — Immune therapeutic response prediction between the high- and low-risk groups. (A) TIDE prediction in the high- and low-risk groups of CRC in TCGA. (B) IPS prediction in the high- and low-risk groups of CRC in TCGA. [file Image_4.tif]

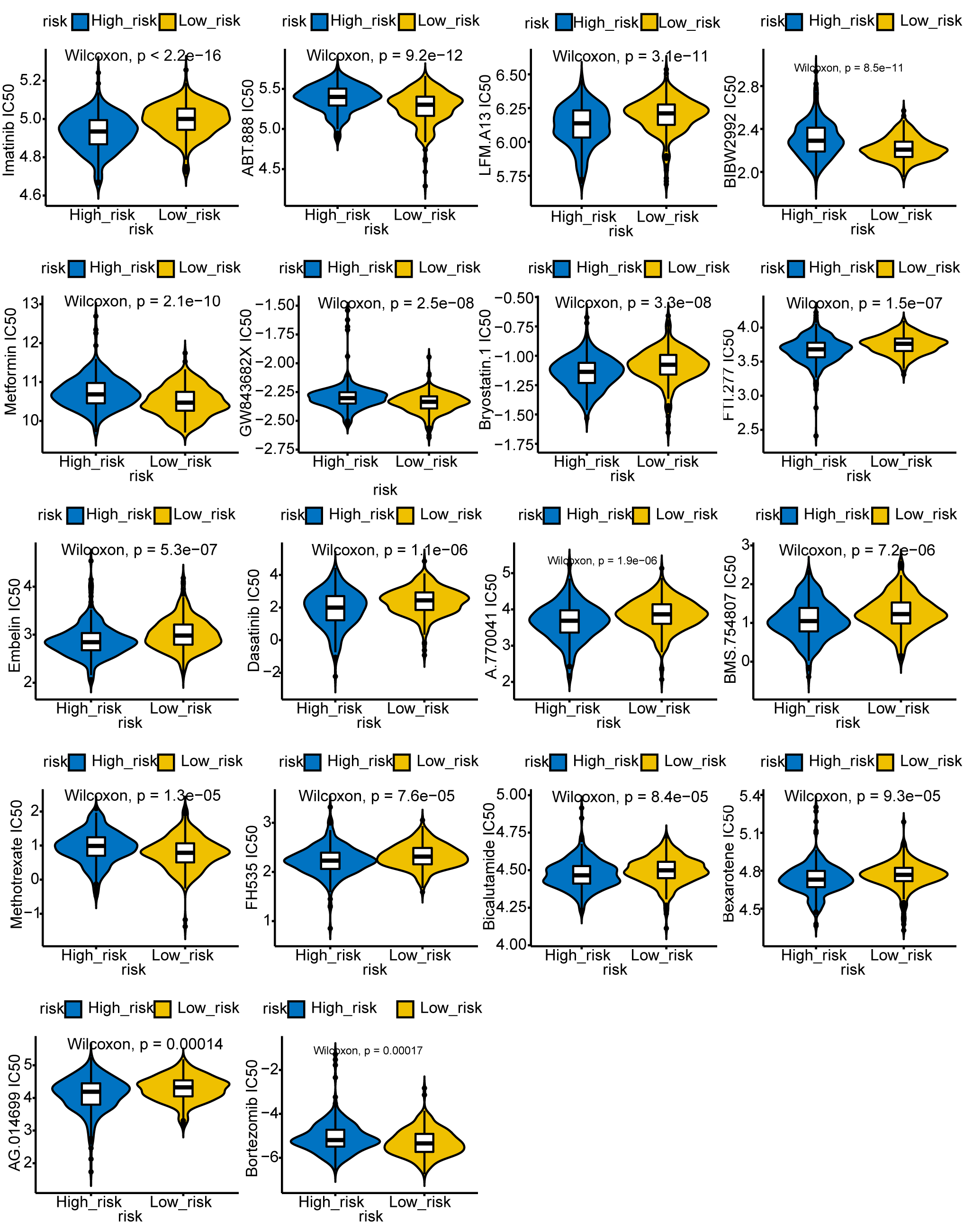

Supplement: Supplementary Figure 5 — Therapeutic response prediction of 33 drugs between the high- and low-risk groups. [file Image_5.tif]
